# Synergistic effects of compost amendment and foliar nano-micronutrients on yield and quality of sugar beet grown in calcareous soil

**DOI:** 10.1038/s41598-026-38845-5

**Published:** 2026-02-27

**Authors:** Ibrahim S. H. El-Gamal, El-Araby S. R. Salem, Hossam M. El-Sharnoby, Amira E. El-Sherief, Noura F. G. Salem, Reda M. Y. Zewail, Ali A. Badawy, Heba S. El-Desouky

**Affiliations:** 1 Sugar Crops Research Institute, Agricultural Research Center, Giza, Egypt; 2 Botany and Microbiology Department, Faculty of Science (Girls Branch), Al-Azhar University, Cairo, 11754 Egypt; 3 Botany Department, Faculty of Agriculture, Benha University, Benha, 13736 Egypt; 4 Botany and Microbiology Department, Faculty of Science, Al-Azhar University, Cairo, 11884 Egypt

**Keywords:** Sugar beet, Calcareous soil, Compost, Nano-micronutrients, Quality, Yield, Sustainability, Nutrient efficiency, Photosynthetic pigments, Antioxidant enzymes, Environmental sciences, Plant sciences

## Abstract

Calcareous soils, characterized by high pH and low nutrient availability, pose significant challenges to sustainable sugar beet production. Combining organic soil amendments with advanced nano-fertilizers may offer a synergistic strategy to enhance crop productivity and quality in such marginal environments. A 2-year field study (2022/2023 and 2023/2024) was conducted using a split-plot design to evaluate the effects of three compost rates (0, 3, and 6 tons acre^−1^) and five foliar nano-micronutrient combinations (control, Fe + Mn, Fe + B, Mn + B, Fe + Mn+B applied at 100 mg L^−1^ each). Growth, physiological, yield, and technological quality parameters of sugar beet were analyzed. The integrated application of 6 tons compost acre^−1^ and the nano Fe + Mn+B mixture resulted in powerful synergistic effects, consistently outperforming all other treatments. This optimal combination maximized photosynthetic pigments (e.g., increased chlorophyll a by 27%), boosted antioxidant enzyme activities (catalase by 29%, peroxidase by 42%), and enhanced growth parameters, leading to a 55% greater leaf area index. Consequently, it achieved the highest sucrose content (up to 19.85%), extracted sugar percentage (17.25%), root yield (27.12 tons acre^−1^), and sugar yield (4.68 tons acre^−1^), representing yield increases of 28–32% and 63–69%, respectively, over the compost-only control. For optimal sugar beet productivity in calcareous soils, an integrated management approach is recommended, consisting of soil amendment with 6 tons of compost per acre supplemented by foliar application of a combined nano-iron, manganese, and boron mixture. This strategy effectively enhances soil health, plant physiological performance, and ultimately, sugar yield and quality, providing a sustainable pathway for cultivation in nutrient-deficient calcareous environments.

## Introduction

Sugar beet (*Beta vulgaris* L.), a member of the Amaranthaceae family, is a cornerstone of global sugar production, ranking second only to sugarcane. It supplies roughly 40% of the world’s sugar. Beyond its sugar-rich roots, sugar beet generates valuable by-products—pulp and molasses that serve as nutritious animal feed and residues that feed into a broad spectrum of industrial, economic, and biochemical processes^1, 2^. This crop can thrive in a variety of soil types. However, under specific conditions, it performs exceptionally well in calcareous soils, which are characterized by a high concentration of calcium carbonate (CaCO_3_). Unfortunately, calcareous soils typically have low productivity due to their poor physicochemical properties, low organic matter content, and limited availability of nitrogen and potassium. Moreover, the high pH in these soils decreased the availability of phosphate, zinc, iron, and other micronutrients, with the exception of molybdenum^3^. To improve these soils, the addition of organic matter is crucial, as it significantly enhances soil aggregation, stability, and overall physical and chemical properties^4, 5^. According to Ref.^6^, incorporating compost into calcareous soils improves their physical structure, enhances nutrient availability, and stimulates microbial populations and activity. This practice also suppresses soil nematodes and pathogens, ultimately translating into higher crop yields.

Nanoparticles, which are atomic or molecular aggregates that range from 1 to 100 nanometers in size, have the potential to significantly benefit plant production by promoting growth and yields^7, 8^. They can serve as a replacement for traditional fertilizer application techniques by delivering nutrients to plants in a controlled and gradual manner^9, 10^. Due to their higher absorption rates and reactivity, nanoparticles can be applied to promote plant growth and development^11, 12^. In this regard, Wittig et al.^13^ demonstrated that strategically using micronutrients such as manganese, zinc, and iron can notably increase the productivity of sugar beet. They found that applying a mixture of nano-sized micronutrients (Fe, Mn, Zn, and B) at a concentration of 200 mg L^− 1^ along with 1% urea improved both yield and quality. Additionally, using micronutrients in nanoparticle form can reduce the need for other micronutrients and nitrogen fertilizers^14, 15^. Also, Salem^16^ found that spraying beet plants with nano boron significantly improved various growth metrics, including increases in leaf area index, photosynthetic pigments, and enzyme activity (peroxidase and catalase). They also observed enhancements in root and sugar yields acre^− 1^, while the alpha-amino nitrogen content and sugar lost to molasses % were reduced compared to other treatments. However, the impact on the quality index, sucrose content, and the quantity of extracted sugar was insignificant. A study of El-manhaly^17^ reported that increasing the nano-fertilization level to 500 ppm significantly enhanced root diameter, foliage weight plant^− 1^, sucrose %, quality index, and sugar yield acre^− 1^. Notably, applying 250 ppm resulted in a substantial increase in root weight and yield acre^− 1^.

This study aimed to evaluate the synergistic effects of compost amendment and foliar nano-micronutrient mixtures on the growth, yield, and quality of sugar beet (*Beta vulgaris* L.) cultivated in calcareous soils under drip irrigation. We aimed to identify the ideal combination of compost and nano-fertilizers to boost photosynthetic efficiency, enhance antioxidant activity, increase both root and sugar yields, and curb sugar losses to molasses. By tackling the high pH and low nutrient availability characteristic of calcareous soils, our goal was to propose practical, sustainable soil-health strategies that maximize sugar beet productivity in these marginal environments.

## Materials and methods

### Experiment layout

Two field experiments were conducted at a private farm in El Gharbaniat, Borg El-Arab (latitude 30.55° N and longitude 29.32° E), Alexandria Governorate, Egypt, in the 2022/2023 and 2023/2024 seasons to find out the effect of organic fertilization and the foliar application of micronutrients as nano-fertilizer on the growth, yield, and quality of sugar beet (*Beta vulgaris* var. *saccharifera* Alef.) under a drip irrigation system in calcareous soil. This study included fifteen treatments consisting of three compost application rates to the soil (zero, 3, and 6 tons acre^− 1^) and spraying five nano-micronutrient mixtures {no spray, (Fe + Mn), (Fe + B), (Mn + B), and (Fe + Mn + B)} and their interactions. A split-plot design with four replicates was used; the three rates of compost were allocated in the main plots, while the five combinations of nano micronutrients were randomly sprayed in the sub-plots. The sub-plot area was 21.6 m^2^ and consisted of six ridges with a length of 6 m and 60 cm in width, with 20 cm between hills. The German multi-germ sugar beet variety “Bts 185” was obtained from the Sugar Crops Research Institute at the Agricultural Research Center of the Ministry of Agriculture in Giza Governorate, Egypt. The seeds were sown in the 1st week of October, while the beets were harvested at 210 days after sowing in both seasons.

The mixed compost, supplied by the Americano Agricultural Development Company (El-Sheikh Zaied, 6 October City, Giza, Egypt), was incorporated into the soil during seedbed preparation. Its analysis was conducted at the Soil Unit of the Agricultural Research Center (ARC) following the methods of Page et al.^18^, with results presented in Table 1. The nanomaterials (Fe, Mn, and B nanoparticles) were sourced from the Nanotechnology Project, Department of Physiology, Faculty of Agriculture, Cairo University, Giza, Egypt. The nanoparticles were in a liquid suspension and exhibited a needle-like morphology, with average diameters of 21 ± 9, 25 ± 8, and 20 ± 7 nm, and average lengths of 87 ± 1, 86 ± 1, and 89 ± 2 nm for Fe, Mn, and B, respectively. A foliar spray of nano-micronutrients (100 mg L^−1^ of each micronutrient) was applied three times: after thinning, followed by two subsequent applications at 20-day intervals. The application volume was 100 L acre^−1^ for the first spray and 200 L acre^−1^ for the second and third sprays.

Plants were thinned at the 4-leaf stage to ensure one plant per hill. Agricultural sulfur was applied during soil preparation as a general treatment at a rate of 300 kg acre^− 1^. Phosphorus was added in the form of phosphoric acid (H_3_PO_4_), which has a density of 1.6 and a purity of 85%. It was applied as fertigation at a rate of 49 kg acre^− 1^, divided into three doses during the first two weeks after sowing. Nitrogen fertilizer was applied as ammonium nitrate (33.5% N) at a rate of 100 kg N acre^− 1^, divided into two equal doses: the first dose after thinning (4-true leaf stage) and the second one month later. Potassium was added in the form of potassium sulfate (48% K_2_O) at a rate of 50 kg, split into two equal doses: during seedbed preparation and with the first nitrogen dose. Other cultivation procedures were carried out as recommended by the Sugar Crops Research Institute, Agricultural Research Center, Giza, Egypt. Soil samples were collected from the experimental site at a depth of 0–30 cm to determine their physical and chemical properties, using the method outlined by A.O.A.C.^19^, as detailed in Table 2.

### Studied traits

At 110 days after sowing, ten plants were randomly collected from the inner rows of each subplot to assess various biochemical, physiological, and agronomic parameters. For biochemical and physiological analysis, antioxidant enzyme activity in leaves was evaluated by determining catalase (CAT) activity using the method of Aebi^20^ and peroxidase activity following Polle et al.^21^. Enzyme activities were expressed as units per gram of protein (U g^− 1^ protein). Hydrogen peroxide (H_2_O_2_) content was quantified in millimoles per gram of fresh weight, while total phenolics content (mg g^− 1^ dry weight) was measured spectrophotometrically at 750 nm using a UV/Vis spectrophotometer (Jenway, England), according to Le Docte^22^ and Singleton et al.^23^. Leaf area index (LAI) was calculated using the disk method described by Watson^24^, with the formula: leaf area per plant (cm^2^)/plant ground area (cm^2^). Photosynthetic pigments, including chlorophyll a, chlorophyll b, and carotenoids (mg g^− 1^ fresh leaf weight), were determined following von Wettstein^24^.

At harvest, another random sample of ten guarded plants was taken from the inner ridges of each subplot to evaluate root quality traits. Quality analysis of fresh sugar beet roots was conducted at the Nile Sugar Factory Laboratory in Buhaira Governorate, Egypt, where sucrose percentage (Pol %) was determined using Le Docte’s^22^ method. Root impurities—potassium, sodium, and alpha amino-nitrogen—were measured in meq/100 g beet; sodium and potassium were quantified using a flame photometer, while alpha amino-nitrogen was assessed via hydrogenation as per Cooke and Scott^25^. Sugar lost to molasses (SLM %) was calculated using Devillers’^26^ equation: SLM = 0.14(Na + K) + 0.25(α–amino N) + 0.5. Extracted sugar percentage (ES %) was derived using Dexter et al.’s^27^ formula: ES% = sucrose % – SLM % – 0.6. Finally, the quality index (QI) was computed using Cooke and Scott’s^25^ equation: QI = extracted sugar % / sucrose %.

**Table 1:** Selected physicochemical properties of the compost used in the study (dry weight basis).

Analysis	Value	Analysis	Value
Cubic meter weight (kg)	820	C/N ratio	1:16
Moisture (%)	17	Total nitrogen %	0.59
pH (1:10)	7.3	Ammonium nitrogen (ppm)	425
EC (1:10) (ds m^− 1^)	1.1	Nitrate nitrogen (ppm)	24
Organic matter %	47.27	Total phosphorus %	0.35
Organic carbon %	9.81	Total potassium %	0.24
Ash %	38.08	Weed seeds	Zero

**Table 2:** Physical and chemical properties of the experimental calcareous soil during the 2022/2023 and 2023/2024 seasons.

Physical properties particle size			Soil chemical properties																	
Sand %	Silt %	Clay %	pH (1:2.5) Soil	E.C ds m^− 1^	O.M. %	CaCO_3_^−2^	Soluble cations (meq L^− 1^)				Soluble anions (meq L^− 1^)				Available macro and micronutrients (mg kg^− 1^ soil)					
Texture: sandy soil						%	Ca^+ 2^	Mg^+ 2^	Na^+^	K^+^	CO_3_^−2^	HCO_3_^−^	Cl^−^	SO_4_^−2^	*N*	*P*	K	Fe	Mn	B
2022/2023 season																				
80.80	4.55	14.65	8.0	1.74	0.83	12.85	6.10	2.19	5.40	0.71	--	4.40	7.1	2.90	30.5	6.16	175.8	3.4	1.2	0.19
2023/2024 season																				
81.20	3.40	15.40	7.9	1.59	0.92	13.40	5.78	2.20	4.82	1.20	--	4.22	7.2	2.58	31.4	6.07	180.0	3.9	1.1	0.22

### Yields

To determine root yield (ton acre^− 1^), the roots from the guarded rows of each sub-plot were harvested, weighed in kilograms, and subsequently converted into tons acre^− 1^. Sugar yield (ton acre^− 1^) was then calculated using the formula: sugar yield/acre = root yield/acre × extracted sugar percentage.

### Statistical analysis

All obtained data were statistically analyzed according to the technique (Co-STATE) computer software package, using analysis of variance (ANOVA) for the split-plot design as published by Gomez and Gomez^28^. The least significant difference (LSD) method was used to test the differences between treatment means at the 5% level of probability as described by Snedecor and Cochran^29^.

## Results

### Leaf pigments

The experimental results in Table 3 demonstrate a powerful synergistic interaction between compost application and foliar nano-micronutrient supplementation for enhancing photosynthetic pigments in sugar beet under calcareous soil conditions. The integrated strategy of combining the highest compost rate (6 tons acre^− 1^) with the triple nano-micronutrient combination (B + Mn+Fe) consistently yielded peak pigment concentrations, establishing it as the optimal treatment. For instance, this combination increased chlorophyll a by 27% (5.21 vs. 4.1) in the second season compared to the 6-ton compost control, while more dramatic synergistic gains were evident at lower compost levels, such as an 84% increase in chlorophyll b (2.37 vs. 1.29) at the 3-ton level in the first season. The nano-combination efficacy clearly followed the order B + Mn+Fe > B + Mn ≈ Fe + B > Fe + Mn > control, with the triple combination’s superiority underscoring the need to address the multiple concurrent micronutrient deficiencies typical of calcareous soils. Notably, foliar application alone (B + Mn+Fe at zero compost) also provided significant mitigation, boosting pigments by 30% for chlorophyll a and 35% for chlorophyll b in the second season compared to the unamended control. While seasonal variation occurred—most strikingly in a 132% increase in carotenoids (1.16 vs. 0.5) at the 6-ton level in the first season—the very low LSD values confirm the statistical robustness of all treatment effects.

**Table 3:** Impact of compost and nano-micronutrients combinations on photosynthetic pigments (mg g^− 1^ fresh leaf weight) of sugar beet in calcareous areas during 2022/2023 and 2023/2024 seasons.

Treatments		Chlorophyll a		Chlorophyll b		Carotenoids	
Compost level (ton acre^− 1^)	Nano-micronutrients combinations	1st season	2nd season	1st season	2nd season	1st season	2nd season
Zero	Without spraying	2.8	3.24	1.07	1.52	0.47	0.6
	Fe + Mn	2.99	2.25	1.05	1.38	0.35	0.58
	Fe + B	3.32	2.86	1.14	1.65	0.49	0.72
	B + Mn	3.35	3.66	1.31	1.72	0.55	0.73
	B + Mn + Fe	3.69	4.21	1.37	2.06	0.56	0.81
3	Without spraying	3.51	3.15	1.29	1.85	0.54	0.85
	Fe + Mn	3.99	3.25	2.05	2.38	0.75	1.08
	Fe + B	3.32	3.86	2.14	2.65	0.89	1.02
	B + Mn	4.35	3.66	2.31	2.72	0.95	1.03
	B + Mn + Fe	4.69	4.21	2.37	3.06	0.96	1.09
6	Without spraying	3.86	4.1	1.44	1.96	0.5	0.81
	Fe + Mn	4.99	4.25	2.05	2.38	1.05	1.08
	Fe + B	4.32	4.86	2.14	2.65	1.09	1.02
	B + Mn	4.35	4.66	2.31	2.72	1.05	1.03
	B + Mn + Fe	4.69	5.21	2.37	2.06	1.16	1.01
LSD at 0.05		0.03	0.06	0.05	0.03	0.08	0.07

### Antioxidant activities

The data from Table 4 reveal a pronounced and statistically significant synergistic enhancement of sugar beet’s antioxidant defense system and secondary metabolism through the integrated application of compost and foliar nano-micronutrients in calcareous soils. The most effective strategy consistently across both seasons was the combination of the highest compost rate (6 tons acre^− 1^) with the triple nano-micronutrient formulation (B + Mn+Fe). This treatment maximized key stress-mitigation parameters: it increased catalase activity by 29% in the first season (2.98 vs. 2.31 in the 6-ton control) and peroxidase activity by 42% (0.97 vs. 0.51). The most dramatic response was observed in total phenolics, which surged by 55% in the first season (287.10 vs. 185.69) and 31% in the second (282.53 vs. 215.43), indicating a substantial boost in the plant’s antioxidant and defensive compound pool. The efficacy of nano-micronutrients followed a clear hierarchy (B + Mn+Fe > B + Mn ≈ Fe + B > Fe + Mn > control), with the triple combination proving critical for comprehensive nutrient support. Notably, compost application alone established a progressively higher baseline for these biochemical parameters, but the foliar nano-nutrients acted as a powerful catalytic trigger, with their impact often multiplicative rather than merely additive—for example, at the 3-ton compost level, the B + Mn+Fe treatment elevated catalase activity by a remarkable 213% over its respective control (3.98 vs. 1.27) in the first season. While some inter-annual variation in the magnitude of response is apparent, the consistently low LSD values affirm the robustness of the treatment effects.

**Table 4:** Impact of mixed compost and nano-micronutrients combinations on catalase activity (U g^− 1^ protein), peroxidase activity (U g^− 1^ protein) and total phenolics (mg g^− 1^ dry weight) of sugar beet in calcareous areas during 2022/2023 and 2023/2024 seasons.

Treatments		Catalase activity		Peroxidase activity		Total phenolics	
Compost level (ton acre^− 1^)	Nano- trace micronutrients combinations	1st season	2nd season	1st season	2nd season	1st season	2nd season
Zero	Without spraying	1.12	1.51	0.34	0.58	154.57	130.75
	Fe + Mn	1.82	1.96	0.51	0.72	174.74	170.72
	Fe + B	1.57	1.83	0.44	0.62	175.34	171.84
	B + Mn	1.68	1.75	0.33	0.56	182.01	181.52
	B + Mn + Fe	1.98	2.15	0.67	0.88	187.10	182.53
3	Without spraying	1.27	1.57	0.40	0.67	172.71	163.97
	Fe + Mn	2.92	2.96	0.71	0.82	194.74	175.72
	Fe + B	2.97	2.83	0.84	0.92	195.34	171.84
	B + Mn	2.98	2.75	0.93	0.96	202.01	181.52
	B + Mn + Fe	3.98	3.15	0.97	0.98	217.10	182.53
6	Without spraying	2.31	2.03	0.51	0.76	185.69	215.43
	Fe + Mn	2.82	2.96	0.91	0.92	274.74	275.72
	Fe + B	2.57	2.83	0.94	1.02	275.34	271.84
	B + Mn	2.68	2.75	0.93	1.06	282.01	281.52
	B + Mn + Fe	2.98	3.15	0.97	1.08	287.10	282.53
LSD at 0.05		0.15	0.17	0.14	0.12	0.61	1.23

### Leaf area index, root diameter, foliage and root fresh weight

The data presented in Table 5 conclusively demonstrates that the integrated application of compost and foliar nano-micronutrients significantly enhances the growth and morphological yield components of sugar beet in calcareous soils, with the most pronounced effects achieved by combining the highest compost rate (6 tons acre^− 1^) with the triple nano-micronutrient combination (B + Mn+Fe). This optimal treatment resulted in remarkable percentage increases across all measured parameters compared to its corresponding compost-only control (6 tons, without spraying). Specifically, it boosted the leaf area index by 55% in the first season (5.75 vs. 3.72) and by 38% in the second (5.20 vs. 3.78), directly supporting greater photosynthetic capacity. Concurrently, root fresh weight increased by 19% (1427.11 vs. 1196.00) and 29% (1477.67 vs. 1141.20) across seasons, while foliage fresh weight saw increases of 71% (846.33 vs. 494.13) and 59% (800.89 vs. 505.20), respectively. Furthermore, root diameter was enhanced by 14% (9.83 vs. 8.63) and 45% (12.63 vs. 8.71), indicating improved root development and potential yield. The synergistic nature of the treatments is evident, as compost application alone progressively improved baseline growth, but the nano-micronutrients—particularly the complete B + Mn+Fe formulation—acted as a powerful catalyst, with efficacy following the established hierarchy (B + Mn+Fe > B + Mn ≈ Fe + B > Fe + Mn). The results are statistically robust (as confirmed by the LSD values) and consistent across seasons.

**Table 5:** Impact of mixed compost rates and nano-micronutrients combinations on leaf area index (cm^2^), root fresh weight/plant (g), foliage fresh weight/plant (g) and root diameter (cm) of sugar beet in calcareous area during 2022/2023 and 2023/2024 seasons.

Treatments		Leaf area index		Root fresh weight		Foliage fresh weight		Root diameter	
Compost level (ton acre^− 1^)	Nano- trace micronutrients combinations	1st season	2nd season	1st season	2nd season	1st season	2nd season	1st season	2nd season
Zero	Without spraying	2.33	3.17	1049.53	954.67	452.20	442.80	6.43	8.66
	Fe + Mn	2.35	2.56	862.89	925.22	369.33	375.89	6.67	9.03
	Fe + B	2.45	2.70	1098.89	1019.11	448.11	470.78	6.79	9.15
	B + Mn	2.60	2.90	1176.78	1040.44	465.78	487.78	6.92	9.28
	B + Mn + Fe	2.75	3.20	1227.11	1077.67	546.33	500.89	6.83	9.63
3	Without spraying	3.44	3.38	1136.07	1029.67	503.73	480.47	6.52	8.66
	Fe + Mn	3.35	3.56	1162.89	1125.22	669.33	575.89	7.67	10.03
	Fe + B	3.45	3.70	1198.89	1179.11	648.11	670.78	7.79	10.15
	B + Mn	3.60	3.90	1276.78	1240.44	665.78	687.78	7.92	10.28
	B + Mn + Fe	3.75	4.20	1327.11	1377.67	746.33	700.89	7.83	11.63
6	Without spraying	3.72	3.78	1196.00	1141.20	494.13	505.20	8.63	8.71
	Fe + Mn	4.35	4.56	1262.89	1225.22	769.33	675.89	8.67	11.03
	Fe + B	4.45	4.70	1298.89	1279.11	748.11	770.78	8.79	11.15
	B + Mn	4.60	4.90	1376.78	1340.44	765.78	787.78	8.92	11.28
	B + Mn + Fe	5.75	5.20	1427.11	1477.67	846.33	800.89	9.83	12.63
LSD at 0.05		0.20	0.04	25.74	20.15	19.05	12.32	0.276	0.407

### Sodium, potassium and alpha-amino N contents

The results from Table 6 demonstrate a significant positive impact of integrated compost and nano-micronutrient management on the root quality parameters of sugar beet, with the most pronounced and consistent benefits observed for potassium (K) and alpha-amino nitrogen content. The optimal treatment—6 tons acre^− 1^ compost combined with the foliar application of B + Mn+Fe nano-micronutrients—maximized the accumulation of these critical components. This strategy increased alpha-amino N by 62% in the first season (2.42 vs. 1.49 meq/100 g in the 6-ton control) and by 70% in the second (2.35 vs. 1.38), indicating a substantial enhancement of the root’s nitrogen metabolism which is closely linked to sugar extraction efficiency. Potassium content was also elevated, showing increases of approximately 15% (4.90 vs. 4.27) and 21% (4.91 vs. 4.07) across the two seasons, respectively, supporting better osmoregulation and sugar transport. The efficacy of nano-micronutrient combinations followed the established hierarchy (B + Mn+Fe > B + Mn ≈ Fe + B > Fe + Mn), reinforcing the necessity of a balanced multi-nutrient approach. While sodium (Na) content showed an increasing trend with treatments, the differences were not statistically significant (LSD: NS), suggesting that the primary quality improvements are driven by the enhancement of beneficial constituents rather than a reduction of this impurity. The synergistic effect is clear, as compost application alone raised baseline levels, but the addition of catalytic nano-micronutrients, particularly the triple combination, triggered further significant gains.

**Table 6:** Impact of mixed compost rates and nano-micronutrients combinations on Na, K and alpha-amino N contents (meq/100 g roots) of sugar beet in calcareous area during 2022/2023 and 2023/2024 seasons.

Treatments		Na		K		Alpha-amino *N*	
Compost rates (ton acre^− 1^)	Nano-trace micronutrients combinations	1st season	2nd season	1st season	2nd season	1st season	2nd Season
Zero	Without spraying	0.81	1.79	3.48	3.10	1.18	1.08
	Fe + Mn	1.09	1.98	4.13	3.17	1.65	1.03
	Fe + B	1.27	2.15	4.31	3.37	1.57	1.27
	B + Mn	1.38	2.31	4.61	3.63	1.46	1.29
	B + Mn + Fe	1.46	2.37	4.80	3.81	1.22	1.05
3	Without spraying	1.14	1.94	4.11	4.11	1.43	1.05
	Fe + Mn	1.19	2.98	4.53	4.17	1.75	1.73
	Fe + B	1.37	3.15	4.61	4.37	1.87	1.87
	B + Mn	1.58	3.31	4.71	4.63	1.96	1.99
	B + Mn + Fe	1.86	3.37	4.80	4.81	2.22	2.05
6	Without spraying	1.26	2.61	4.27	4.07	1.49	1.38
	Fe + Mn	1.49	3.18	4.73	4.67	1.85	1.83
	Fe + B	1.57	3.35	4.81	4.77	1.97	1.99
	B + Mn	1.68	3.41	4.91	4.93	2.16	2.19
	B + Mn + Fe	1.96	3.67	4.90	4.91	2.42	2.35
LSD at 0.05		NS	NS	0.20	0.19	0.10	0.13

### Sucrose, sugar lost to molasses percentages and quality index

The data from Table 7 conclusively demonstrates that the integrated application of compost and foliar nano-micronutrients significantly enhances the final technological quality of sugar beet roots in calcareous soils, with the optimal strategy being the combination of 6 tons acre^− 1^ compost and the triple nano-micronutrient formulation (B + Mn+Fe). This treatment maximized the crucial sucrose content, increasing it by 26.7% in the first season (19.63% vs. 15.49% in the 6-ton control) and by 26.2% in the second season (19.85% vs. 15.73%), representing a direct and substantial gain in the primary yield parameter. Concurrently, the overall processing quality, as reflected by the quality index, was also improved, rising by 3.4% in the second season (87.16 vs. 84.29). A notable and consistent trend across treatments was a parallel increase in sugar loss to molasses (e.g., + 47.5% and + 75.5% in the first and second seasons under the optimal treatment, respectively), which is likely attributable to the associated elevation of alpha-amino N and potassium impurities as shown in previous data; however, the net gain in sucrose percentage and quality index confirms that the benefits of enhanced growth and metabolism outweigh the increase in these impurities. The synergistic effect is clear, as both compost and nano-micronutrients—particularly the complete B + Mn+Fe combination—are essential for driving these quality parameters to their peak, with efficacy following the established hierarchy.

**Table 7:** Impact of mixed compost rates and nano-micronutrients combinations on sucrose (%), sugar loss to molasses (%), and quality index of sugar beet in calcareous area during 2022/2023 and 2023/2024 seasons.

Treatments		Sucrose		Sugar loss to molasses		Quality index	
Compost rates (ton acre^− 1^)	Nano-trace micronutrients combinations	1st season	2nd season	1st season	2nd season	1st season	2nd Season
Zero	Without spraying	14.53	14.16	1.38	1.42	86.07	82.59
	Fe + Mn	15.44	16.53	1.56	1.48	85.05	83.57
	Fe + B	16.17	16.76	1.64	1.54	85.23	84.61
	B + Mn	16.45	16.89	1.73	1.64	84.86	85.40
	B + Mn + Fe	17.03	17.05	1.79	1.70	85.03	86.44
3	Without spraying	14.55	15.36	1.63	1.49	85.62	85.53
	Fe + Mn	16.55	16.24	1.78	1.72	85.83	85.75
	Fe + B	17.17	17.25	1.86	1.88	85.86	86.63
	B + Mn	18.17	17.87	1.94	2.14	86.46	86.62
	B + Mn + Fe	18.44	18.22	2.13	2.24	86.69	86.78
6	Without spraying	15.49	15.73	1.58	1.39	86.08	84.29
	Fe + Mn	17.58	17.84	1.98	1.82	86.81	85.81
	Fe + B	18.46	18.72	2.16	1.98	86.69	85.88
	B + Mn	19.15	19.51	2.24	2.34	86.55	84.89
	B + Mn + Fe	19.63	19.85	2.33	2.44	86.66	87.16
LSD at 0.05		0.18	0.30	0.07	0.12	0.17	0.32

### Extracted sugar, root and sugar yields

The data from Table 8 provide the definitive agronomic and economic validation of the experiment, demonstrating that the integrated application of compost and foliar nano-micronutrients culminates in dramatic improvements in the final yield parameters of sugar beet. The synergistic combination of 6 tons acre^− 1^ compost and the B + Mn+Fe nano-micronutrient formulation proved optimal, delivering the highest extracted sugar percentage, root yield, and most critically, sugar yield per acre. This treatment increased extracted sugar by 27.7% in the first season (17.19% vs. 13.46% in the 6-ton control) and by 27.5% in the second (17.25% vs. 13.53%). The enhancements in root yield were equally substantial, at 32.4% (27.11 vs. 20.48 tons acre^− 1^) and 28.3% (27.12 vs. 21.14 tons acre^− 1^) across seasons. Consequently, the paramount parameter of sugar yield exhibited remarkable gains of 68.8% (4.66 vs. 2.76 tons/acre) and 63.6% (4.68 vs. 2.86 tons acre^− 1^), directly translating the physiological and quality benefits observed earlier into superior economic output. The clear efficacy hierarchy of nano-micronutrient combinations (B + Mn+Fe > B + Mn > Fe + B > Fe + Mn) and the progressive improvement with compost level underscore the complementary roles of soil amendment and foliar nutrition.

**Table 8:** Impact of mixed compost rates and nano-micronutrients combinations on extracted sugar (%), root yield (ton acre^− 1^), and sugar yield (ton acre^− 1^) of sugar beet in calcareous area during 2022/2023 and 2023/2024 seasons.

Treatments		Extracted sugar		Root yield		Sugar yield	
Compost level (ton acre^− 1^)	Nano- micronutrients combinations	1st Season	2nd Season	1st season	2nd season	1st season	2nd Season
Zero	Without spraying	12.61	12.33	16.47	16.67	2.08	2.05
	Fe + Mn	13.29	14.68	21.26	20.05	2.83	2.94
	Fe + B	13.97	14.75	21.49	20.36	3.00	3.00
	B + Mn	14.15	14.84	22.46	20.67	3.18	3.07
	B + Mn + Fe	14.69	14.94	22.43	21.11	3.29	3.16
3	Without spraying	12.56	13.35	18.65	20.89	2.34	2.79
	Fe + Mn	14.37	14.18	22.24	21.78	3.19	3.09
	Fe + B	14.90	15.16	23.79	22.18	3.54	3.36
	B + Mn	15.82	15.75	24.36	22.69	3.85	3.57
	B + Mn + Fe	16.05	16.04	25.41	23.12	4.08	3.71
6	Without spraying	13.46	13.53	20.48	21.14	2.76	2.86
	Fe + Mn	15.42	15.64	24.51	24.35	3.78	3.81
	Fe + B	16.22	16.38	25.43	25.34	4.12	4.15
	B + Mn	16.81	16.96	26.17	27.48	4.40	4.66
	B + Mn + Fe	17.19	17.25	27.11	27.12	4.66	4.68
LSD at 0.05		0.18	0.20	0.50	0.42	0.27	0.19

## Discussion

Sugar beet (*Beta vulgaris* L.) is one of the most important sugar crops globally, particularly due to its high productivity in calcareous soils, which are widespread in many arid and semi-arid regions^1, 2^. However, the high pH and poor physical and chemical properties of calcareous soils severely limit the availability of nutrients, particularly essential micronutrients such as iron, manganese, and boron, limiting yield and quality^3^. To address these limitations, organic fertilizer amendment has been chosen as a key strategy. It has been shown to improve soil structure, increase organic matter content, enhance nutrient retention and availability, and stimulate beneficial microbial activity, thus improving the difficult conditions of calcareous soils^6^. Complementing this soil fertilization approach, we incorporated foliar application of nanoscale micronutrients to overcome the unique challenges of low nutrient solubility and uptake in high pH environments. Thanks to their high surface area-to-volume ratio, enhanced reactivity, and improved uptake efficiency, nanoparticles provide an excellent delivery mechanism for precise and efficient micronutrient transport, helping to overcome soil limitations^10, 11^. This integrated strategy, combining the soil health benefits of compost with nanoscale fertilization, represents a novel and sustainable approach to maximize sugar beet productivity in marginal calcareous environments.

The enhancement in photosynthetic pigments from compost applications can be attributed to the organic material in the compost, which likely improved the soil’s water-holding capacity. As a result, beet plants were able to absorb more nutrients and water, leading to a higher concentration of chlorophyll (a and b). It was recorded that different formulated compost applications significantly enhanced leaf pigment contents of lupine^30^. Moreover, Koné et al.^31^ suggested that nutrients present in different types of compost, especially organic molecules with specific chemical properties such as humic acids or phenolic compounds, may play a role in improving plant growth conditions. The increases in photosynthetic pigments from nano-micronutrients may be attributed to the synergistic effects of the three applied nano-micronutrients. This finding is consistent with those investigated by Salem^16^, who reported that spraying beet tops with 100 ppm of nano-micronutrients (Fe, Mn, and B) significantly enhanced the levels of photosynthetic pigments compared to untreated plants. Additionally, it has been reported that micronutrients play a vital role in crop growth and are involved in the production of pigments and hormones, improve other nutrient uptake, respiration, and nitrogen metabolism, and increase plant photosynthetic efficiency^32, 33^. These minerals in the form of nanoparticles play a vital role in improving the germination rate of seedlings, including physiological and biochemical activities such as photosynthesis, antioxidant enzyme activity, chlorophyll quality, carbohydrate content, and plant productivity, as the properties of these particles are different from those in the bulk form^34^.

The observed significant increase in chlorophylls and carotenoids (Table 3) under the combined treatment (6 tons compost + nano Fe + Mn+B) can therefore be attributed to a dual mechanism. First, the compost-improved soil health provides a better foundation of water, nitrogen, and other resources necessary for the biosynthesis of photosynthetic pigments and proteins^30^. Second, the nano-micronutrients directly supply the limiting metallic cofactors required for chlorophyll synthesis (Fe) and for the stabilization and function of pigment-protein complexes (Mn)^34^. This is supported by Salem^16^, who reported that spraying nano-micronutrients significantly enhanced pigment concentrations. The synergy is evident, as the compost-amended soil likely supports a larger, healthier canopy (evidenced by increased LAI, Table 5), which, when supplied with optimal levels of bioavailable micronutrients, achieves maximal photosynthetic efficiency. Furthermore, the combination mitigates oxidative stress, which can otherwise impair photosynthesis. The nano-micronutrients, particularly Fe and Mn, are known to activate antioxidant enzymes like catalase and peroxidase (Table 4)^35, 36^. The compost, by promoting overall plant vigor, also enhances the plant’s innate defense systems. This reduction in reactive oxygen species (ROS) protects the thylakoid membranes and photosynthetic proteins from damage, ensuring sustained photosynthetic performance under potential stress conditions^34, 36^.

The significant enhancement in the activities of catalase (CAT) and peroxidase (POD), along with increased total phenolic content in sugar beet leaves following compost application (Table 4), can be attributed to compost-induced improvements in soil-plant system dynamics and a consequent reduction in physiological stress. In calcareous soils, high pH and poor nutrient availability can induce oxidative stress in plants, leading to the accumulation of reactive oxygen species (ROS) such as hydrogen peroxide (H_2_O_2_) ^3^. Antioxidant enzymes like CAT and POD form the first line of defense, scavenging H_2_O_2_ and other peroxides to protect cellular integrity^20, 21, 37^. The application of compost mitigates the primary abiotic stressors by improving soil physical structure, enhancing water retention, and increasing the availability of essential nutrients and beneficial microbes^6, 30^. This improved rhizosphere environment reduces the metabolic burden associated with nutrient and water deficiency, thereby lowering baseline ROS production. Furthermore, compost is a rich source of organic compounds, including humic and fulvic acids, which have been shown to possess bio-stimulatory properties. These compounds can directly enhance plant metabolism and are known to upregulate the synthesis and activity of key antioxidant enzymes as part of a systemic acquired acclimation response^31, 38^. The study by Koné et al.^31^ supports the role of compost-derived bioactive molecules in eliciting plant defense mechanisms. The observed increase in total phenolic compounds is a complementary non-enzymatic antioxidant response. Phenolics act as potent free-radical scavengers and metal chelators, providing a crucial secondary layer of protection^39^. The improved nutrient status and soil health fostered by compost amendment, particularly the enhanced availability of micronutrients that serve as cofactors for phenylalanine ammonia-lyase (PAL), a key enzyme in the phenylpropanoid pathway, likely drive the biosynthesis of these protective phenolic compounds^40^. This integrated bolstering of both the enzymatic (CAT, POD) and non-enzymatic (phenolics) antioxidant systems equips the plant with a more robust capacity to manage oxidative stress. This leads to greater cellular stability, preserved photosynthetic function—as oxidative damage to chloroplasts is minimized—and ultimately supports sustained growth and higher yield potential, as evidenced by the improved growth metrics and yield in compost-treated plots (Tables 5 and 8)^36, 41^. The advantage of applying nano-micronutrients is their superior ability to penetrate plant cells, which enhances nutrient uptake efficiency compared to conventional micronutrient forms. Nanoparticles stimulate various physiological and enzymatic processes within the plant, notably catalase and peroxidase, which are crucial for mitigating oxidative stress by reducing the accumulation of reactive oxygen species (ROS). Moreover, the increase in phenolic compounds enhanced natural defense mechanisms, which contributed to improved plant growth and stress tolerance. Therefore, the foliar application of nano-micronutrients, especially the combination of iron, manganese, and boron, activates essential biological processes within the plant, leading to enhanced photosynthetic and metabolic efficiency. This, in turn, has a positive impact on crop quality and yield. These findings align with those reported by Ghazy et al.^35^, who found that the application of nano-iron, manganese, or boron significantly influenced the activities of catalase and peroxidase, as well as total phenolic levels compared to unsprayed beets. Additionally, it has been submitted that several metals in the nano form boosted the activities of antioxidant systems in different crops^36^.

The positive effect of compost on growth traits can be attributed to its ability to temporarily lower soil pH, which improves the availability of certain nutrients, as well as its enhancement of the soil’s physical properties that promote root development. These findings align with those reported by Marajan et al.^41^, who noted that applying 5 tons of compost significantly improved LAI, root diameter, and fresh weight compared to the check treatment. Compost positively influences plant growth and yield, likely due to the presence of beneficial metabolites that support nutrient uptake, stimulate growth, and enhance the metabolism of photosynthates, ultimately boosting both productivity and crop quality^30^. When the substance is prepared as nanoparticles, water solubility, dissolution rate, and diffusion uniformity are significantly increased upon administration. These alterations collectively improve plant growth and development^42^. The marked improvement in growth traits—including leaf area index, root diameter, and fresh biomass—following foliar nano-micronutrient (Fe, Mn, B) application stems from the enhanced bioavailability and physiological efficiency afforded by the nano-form. Owing to their high surface area and unique physicochemical properties, nano-fertilizers enable superior foliar uptake, mobility, and systemic delivery compared to conventional forms, ensuring these nutrients reach critical metabolic sites^9, 11^. Each micronutrient plays a distinct yet synergistic role: Fe is essential for chlorophyll synthesis and electron transport, boosting photosynthetic capacity^32^; Mn acts in the oxygen-evolving complex of Photosystem II and activates enzymes central to nitrogen and carbon metabolism, linking light capture to growth^33, 43^; and B strengthens cell wall structure and facilitates sugar translocation, promoting meristematic activity and assimilate partitioning^16^. Together, they enhance photosynthetic output, support cell expansion and division, and mitigate oxidative stress via improved antioxidant activity, collectively driving the accumulation of root and foliage biomass^34, 36^. Integrating 6 tons of compost with foliar spraying of nano-micronutrients (B + Mn+Fe) represents an effective strategy for enhancing sugar beet growth traits and productivity. This combination optimizes both soil health and plant metabolic efficiency^44^. The study also reported that the nano-micronutrient forms are essential micronutrients required in small amounts by crop plants. They play vital roles in various metabolic and physiological processes within the plant, activating enzymes and regulating the metabolism of carbohydrates and proteins, which are critical for the development and differentiation of plant cells.

The results for alpha-amino N from compost may be attributed to the promotional effects of compost on plant growth, achieved through a reduction in rhizosphere pH. This reduction enhances nutrient availability, leading to improved absorption and translocation of nutrients within the plant. Similar results were reported by Nassef et al.^45^, who pointed out that compost applications to new reclaimed soils of Egypt reduced soil pH and its content of CaCO_3_ and increased soil cation exchange capacity (CEC) and its content of both OM and available macro- (N, P, K, S, Ca, and Mg) and micronutrients (Fe, Mn, Zn, and Cu). Moreover, organic fertilization was shown to improve soil health significantly by increasing its nitrogen, organic matter, and humus content; enhancing its structure and water-holding capacity; and accelerating microbial decomposition to release essential nutrients^38^. The results for potassium and alpha-amino N from nano-micronutrients may be explained by the synergistic effect arising from essential micronutrients and bioactive compounds in the nano-combination. These results align with Malvi^43^, who reported that potassium has a direct synergistic relationship with iron and manganese, both of which play a crucial role in chlorophyll formation. Moreover, nano-fertilizers are designed with smart nanodevices that release nitrogen and phosphorus in sync with the plant’s needs. This targeted release not only minimizes nutrient losses to the environment but also prevents unwanted interactions with soil, water, and air^46^.

The improvements in quality parameters from compost can be attributed to better conditions in the rhizosphere resulting from the application of compost, which helps retain soil moisture over the long term and enhances the availability of nutrients for sugar beet plants. Compost at 6 tons per acre significantly enhanced sugar beet quality by increasing sucrose %, reducing SLM%, and improving the QI. Improved synchronization is associated with increased efficiency in plant resource use, resulting in a synergistic effect that positively impacts yield, often exceeding levels observed in untreated plants^40^. Nano-micronutrients, particularly B + Mn + Fe, notably enhanced sucrose content and higher impurity levels as shown previously (Table 7), which in turn raised SLM % and could challenge sugar crystallization during processing. A balance must be achieved between yield-enhancing treatments such as (B + Mn + Fe) and those that reduce impurities like (Fe + Mn or Fe + B) to maximize sugar recovery and industrial processing efficiency. By acting as catalysts, some nanoparticles have shown great potential in enhancing agricultural productivity, including improving crop growth, increasing yields, and increasing biomass production^47, 48^. The interaction between compost and nano-micronutrients is critical for improving sugar beet quality. The combination of (B + Mn + Fe), particularly when used with 6 tons of compost, offers a promising strategy to optimize both sucrose content and quality index. These findings support the use of integrated nutrient management approaches that blend organic amendments with precision foliar applications to enhance sugar beet performance and processing efficiency. A similar result for yields from compost was reported by Manirakiza and Şeker^49^, who stated that compost application appreciably improved soil fertility by enriching pH, electrical conductivity, extractable cations, available micronutrients, and organic matter. It was also documented that nano-nutrients promote the plant’s ability to absorb water and nutrients, subsequently improving photosynthesis and the production of sugars and other biological components^50^.

Finally, supplying sugar beet plants with 6 tons of compost along with spraying their canopies with a mixture of nano boron+manganese+iron resulted in the highest extracted sugar %, root, and sugar yields per acre, compared with the other combinations studied. Treatments with only one or two micronutrients (e.g., Fe + Mn or Fe + B) had moderate effects compared to the full combination. This suggests that a balanced micronutrient supply is essential, especially under stress conditions such as high pH soils.

## Conclusion

This two-year study conclusively demonstrates that the synergistic integration of 6 tons of compost per acre with foliar application of nano Fe + Mn+B represents a transformative agronomic strategy for sugar beet cultivation in calcareous soils. The combined treatment consistently delivered the highest performance, translating improved physiological function into remarkable gains in yield and quality. Specifically, it achieved a 63–69% increase in sugar yield (4.66–4.68 vs. 2.76–2.86 tons acre^− 1^ in the 6-ton compost control), driven by a 28–32% boost in root yield (27.11–27.12 vs. 20.48–21.14 tons acre^− 1^) and a 27% increase in extracted sugar percentage (17.19–17.25% vs. 13.46–13.53%). The enhancement in final product quality was underscored by a 26% rise in sucrose content (19.63–19.85% vs. 15.49–15.73%). These agronomic triumphs were rooted in superior plant physiology: a 27% increase in chlorophyll a, a 55% surge in leaf area index, and a 29–42% enhancement in key antioxidant enzymes (catalase and peroxidase). The results validate a powerful synergy where compost ameliorates the soil environment, while nano-micronutrients precisely correct multiple nutrient deficiencies, acting as a catalytic booster. For sustainable and profitable sugar beet production in calcareous regions, this integrated soil-foliar management approach is therefore unequivocally recommended.

## Data Availability

The datasets generated and/or analyzed during the current study are available from the corresponding author upon reasonable request. To clarify, the study did not involve human or animal samples.
